# Prospective associations of family conflict with alcohol expectancies in the adolescent brain cognitive development study: effects of race and ethnicity

**DOI:** 10.3389/fpsyt.2024.1250351

**Published:** 2024-03-14

**Authors:** Skye C. Bristol, Micah E. Johnson, Wesley K. Thompson, Matthew Albaugh, Alexandra Potter, Hugh Garavan, Nicholas Allgaier, Masha Y. Ivanova

**Affiliations:** ^1^ Department of Mental Health Law and Policy, College of Behavioral and Community Sciences, University of South Florida, Tampa, FL, United States; ^2^ Laureate Institute for Brain Research, Tulsa, OK, United States; ^3^ Department of Psychiatry, Robert Larner College of Medicine, University of Vermont, Burlington, VT, United States

**Keywords:** alcohol, alcohol expectancies, family conflict, race and ethnicity, ABCD study

## Abstract

**Introduction:**

Alcohol expectancies predict subsequent alcohol use and related problems among adolescents, although predictors of alcohol expectancies remain unclear. This study examined the longitudinal association between family conflict, a sociocultural factor strongly implicated in adolescent alcohol use, and positive and negative alcohol expectancies of adolescents of diverse racial/ethnic backgrounds.

**Methods:**

Data were from the Adolescent Brain Cognitive Development Study 4.0 release, a multisite longitudinal study (N = 6,231, baseline age 9–10). Linear mixed-effects regression, with interactions between race/ethnicity and family conflict, tested the association between family conflict and alcohol expectancies, for each racial/ethnicity (e.g., Black vs. non-Black; White vs. non-White).

**Results:**

Interactions of family conflict with race/ethnicity in predicting negative and positive alcohol expectancies were statistically significant for models testing Black and White adolescents, but not for Asian, Hispanic, and Other. Family conflict at baseline predicted lower negative alcohol expectancy for Black adolescents (*B* = −.166, *p* = 0.033) and positive alcohol expectancy for White adolescents (*B* = 0.71, *p* = 0.023) at the year 3 follow-up. All models controlled for sex, age, family socioeconomic status, alcohol expectancies at year 1, and family conflict at year 3.

**Conclusion:**

The results indicate that family conflict is a potential risk factor for problematic alcohol expectancies for Black and White adolescents. Although we did not directly compare Black and White adolescents, our findings indicate that family conflict may operate differently for Black and White adolescents. Prevention and intervention efforts targeting family conflict may be relevant for different aspects of alcohol expectancies in Black and White families.

## 1 Introduction

Alcohol is the most commonly used substance among adolescents and a major public health concern in the United States (1). Approximately 1.8 million adolescents ages 12–17 initiated alcohol use in 2021 (2), and 36.3% of all 8th, 10th, and 12th graders reported lifetime alcohol use in 2019 (3). Alcohol use among adolescents increases the likelihood of alcohol-related problems and psychiatric disorders into adulthood (4, 5). It is also associated with adverse developmental outcomes such as social problems, misuse of other substances, and alterations in brain development with long-term effects (6–8).

Alcohol expectancies are a key predictor of future alcohol use and related problems among adolescents (9–11). They refer to an individual’s anticipated outcomes of alcohol consumption. Alcohol expectancies include negative expectancies, or negative anticipated outcomes (e.g., legal trouble, health problems), and positive expectancies, or positive anticipated outcomes (e.g., feeling more relaxed and joyful). Negative expectancies deter, and positive expectancies motivate adolescent alcohol consumption (9, 12). For example, higher levels of positive alcohol expectancies are associated with early initiation of alcohol use and future alcohol use disorder for adolescents (13–16). However, higher levels of negative alcohol expectancies are associated with lower alcohol consumption for adolescents (13, 17).

There is a growing body of evidence suggesting that alcohol expectancies operate differently for adolescents of different racial and ethnic backgrounds. For example, Meier and colleagues (18) found that adolescents of different racial and ethnic backgrounds reported different levels of alcohol expectancies, while controlling for grade and gender. They also found that positive alcohol expectancies were associated more strongly with drinking frequency and binge drinking for Black than White adolescents, and with drinking initiation for White than Black adolescents (18). Banks and Zapolski (19) found no significant differences in alcohol expectancies or consumption between Black and White adolescents; however, positive alcohol expectancy predicted alcohol use in White but not Black adolescents.

Banks and Zapolski recommended investigating sociocultural factors to understand racial and ethnic differences in alcohol expectancies during adolescence (19). Sociocultural environments shape adolescents’ attitudes toward substance use, with parental level of educational attainment, adverse childhood experiences, and both parental and peer attitudes toward alcohol and alcohol consumption found to be associated with adolescent alcohol expectancies (20, 21). Adolescents of different racial and ethnic backgrounds experience different sociocultural environments, which may explain racial/ethnic differences in alcohol expectancies. For example, the Minority Family Stress Model (22–24) posits that adolescents from ethnically and racially minoritized backgrounds form expectancies about alcohol in the context of greater family stress in comparison to their White peers due to endemic and systemic racism. Greater family stress in families of minoritized backgrounds and the associated alcohol use to cope with such stress may result in adolescents in these families developing expectancies of alcohol as a coping mechanism.

One sociocultural factor, family conflict, has been strongly linked to adolescent alcohol use and expectancies. Family conflict has been associated with the initiation and sustained use of alcohol (25–28). Family conflict was also found to mediate the influence of adolescents’ impulsivity on alcohol use (29). As for alcohol expectancies, Swaim and Stanley (30) found that in a sample of American Indian students, positive alcohol expectancies mediated the relationship of family conflict and alcohol use for female, but not male students (negative expectancies were not assessed in this study). Overall, the well-established association between family conflict and adolescent alcohol use and the emerging evidence linking family conflict and positive alcohol expectancies for American Indian female adolescents suggest that the association between family conflict and alcohol expectancies is worth exploring.

Overall, there is a robust body of empirical evidence indicating that alcohol expectancies are significant contributors to adolescent alcohol use (9–11, 19). There is also emerging evidence that alcohol expectancies operate differently for different ethnic and racial groups (18, 19). It is critically important to understand these differences to develop culturally relevant prevention and intervention programs targeting expectancies for adolescents of different ethnic and racial backgrounds. Examining how sociocultural factors affect alcohol expectancies for different racial/ethnic groups may inform the understanding of the differential operations of alcohol expectancies for adolescents of different ethnic and racial backgrounds (19). Additionally, it may help inform culturally relevant prevention and intervention efforts for racially and ethnically minoritized adolescents.

This study aimed to test the association of family conflict and alcohol expectancies among adolescents of diverse racial and ethnic backgrounds in the Adolescent Brain Cognitive Development (ABCD) Study cohort. We estimated the effects of family conflict assessed at year 1 and alcohol expectancies at year 3 follow-up (when the children were 12–13 years old), while controlling for initial levels of alcohol expectancies, family conflict at year 3, and key demographic variables at baseline.

Our main research questions were (*1) Does family conflict at baseline predict positive and negative alcohol expectancies at year 3 follow-up, while controlling for positive and negative alcohol expectancies at baseline, age, sex, and family SES*, and *(2) Does the association between family conflict at baseline and positive and negative alcohol expectancies at year 3 follow-up vary by adolescents’ race and ethnicity, while controlling for the same covariates?* Based on the Minority Family Stress Model (22–24), we hypothesized that due to the unique stressors associated with race/ethnicity in families of minoritized racial and ethnic backgrounds, (1) adolescents from minoritized racial and ethnic backgrounds would report higher levels of family conflict than White adolescents, and (2) family conflict would have stronger associations with problematic alcohol expectancies (i.e., higher positive and lower negative) for adolescents of minoritized racial and ethnic backgrounds than for White adolescents.

## 2 Methods

### 2.1 Data

We conducted a secondary longitudinal analysis using data from the Adolescent Brain Cognitive Development (ABCD) Study (4.0 release), a longitudinal cohort study of 11,880 adolescents ages 9–10 years old at baseline from 21 data collection sites across the United States (31). A probability sampling strategy was employed to identify schools within the communities surrounding each data collection site, with a small portion of study participants being recruited from non-school-based community outreach and word-of-mouth referrals. The ABCD study follows adolescents and their families from preadolescence into adulthood, tracking biological and behavioral development. Data collection for the baseline cohort began in September 2016 and ended in October 2018, with annual lab-based assessments and biennial imaging acquisition follow-ups. Written assent was obtained from adolescents, and written informed consent was obtained from parents or guardians.

### 2.2 Sample

This study included adolescents with data on family conflict measured at and alcohol expectancy measured at year 1 and year 3 follow-ups, when the children were 12 to 13 years old. COVID-19 restrictions in March 2020 required remote assessments when in-person collection was not permitted or feasible, affecting the 2- and 3-year follow-up assessments and the 30-month assessments conducted from March 2020 on. The total sample was 6,231 adolescents.

### 2.3 Measures

#### 2.3.1 Alcohol expectancies

Alcohol expectancies were assessed using the Alcohol Expectancy Questionnaire-Adolescents, Brief (AEQ-AB), based on the AEQ-A and measures two factors of general positive and general negative. The AEQ-AB has good internal consistency and demonstrated validity in predicting adolescent drinking patterns (32). The 7-item questionnaire is a 5-point Likert scale (1 = disagree strongly to 5 = agree strongly) on adolescents’ expectancies about the effects of alcohol. The AEQ-AB includes statements such as “Alcohol helps a person relax, feel happy, feel less tense, and can keep a person’s mind off of mistakes at school or work” and “Alcohol can help how well a person gets along with others (makes people want to have fun together)”. The AEQ-AB is composed of two components: general positive (AEQ-ABp) (items 1,2,4,6) and general negative (AEQ-ABn) (items 3,5,7). A summary score was calculated for positive and negative alcohol expectancies (see **Table 1**). The scores collected at year 3 follow-up were used for the outcome variable of this study.

**Table 1:** Alcohol expectancy questionnaire (AEQ)adolescent, brief items.

General Positive (AEQ-ABp)	General Negative (AEQ-ABn)
Item 1. Alcohol helps a person relax, feel happy, feel less tense, and can keep a person’s mind off of mistakes at school or work.	Item 3. Alcohol can hurt how well a person gets along with others (makes people mean to others).
Item 2. Alcohol can help how well a person gets along with others (makes people want to have fun together).	Item 5. Alcohol hurts how people think and it hurts their coordination (run into things, act silly, have a hangover).
Item 4. Alcohol helps people think better and helps coordination (people understand things better; can do things better).	Item 7. Alcohol can make people more careless or do things that could get them into trouble (do things they feel bad about; do things they regret).
Item 6. Alcohol makes a person feel stronger and more powerful (easier to fight, speak in front of others, stand up to others).	

The summary score for positive alcohol expectancy includes items 1, 2, 4, and 6. The summary score for negative alcohol expectancy includes items 3, 5, and 7.

#### 2.3.2 Family conflict

Family conflict was assessed with the Family Conflict subscale of the Family Environment Scale (FES) (33). We used FES completed by adolescents at year 1 because AEQ-AB was not collected until year 1 follow-up. The FES family conflict subscale assesses expressed anger and aggression among family members, composed of nine yes or no questions (e.g., “We fight a lot in our family”; “Family members sometimes get so angry they throw things”). Answers to the questions are summed into a total score, with a higher score indicating higher family conflict.

#### 2.3.3 Race and ethnicity

The definition of race and ethnicity have evolved overtime and utilized as descriptive variables in research to represent social experiences. Researchers have argued that the terms are social constructs and should not be used synonymously (34–36). Race has most often referred to shared physical traits and cultural patterns, whereas ethnicity refers to commonality in culture through shared origins, beliefs, religion, language, and traditions (37). We utilize a simplified version of the race/ethnicity variable and acknowledge the distinction between the two concepts and will reference it to indicate and/or. Parents reported children’s race/ethnicity using the following categories: Asian, Non-Hispanic Black, Hispanic, Non-Hispanic White, and Other (including adolescents that identify as American Indian/Alaska Native (AIAN), Native Hawaiian/Pacific Islander (NHPI), or with two or more races/ethnicities). Binary dummy variables were created as moderators reflecting participants status on the variables (e.g., Asian: yes = 1, no = 0; Black: yes = 1, no = 0). Race and ethnicity at baseline assessment followed the definitions of the NIH Minimum Reporting guidelines and the Office of Management and Budget (OMB) standards (NIH Office of Research on Women’s Health; 38). The ABCD baseline sample closely matches the American Community Survey (ACS)-based national estimates for race and ethnicity (39). However, Asian is underrepresented in the ABCD raw data and sample for this study (2.2%) compared with the ACS national estimates (5.9%), potentially due to differences in reporting multiple race and ethnicity ancestry (38, 40).

#### 2.3.4 Covariates

Covariates in all models included sex assigned at birth, age (in months), family socioeconomic status (SES), adolescent alcohol expectancy, and family conflict. The demographic variables were assessed at baseline, whereas alcohol expectancy was assessed at year 1 and family conflict at year 3. Family SES was operationalized as an ordered categorical variable of the highest level of parental educational attainment for the parent or caregiver enrolled in the study: 1 = less than HS Diploma, 2 = HS Diploma/GED, 3 = Some college, 4 = Associate, 5 = Bachelor, and 6 = Post Graduate Degree. Partner educational attainment was collected, but not included in this study. While SES is a multidimensional construct that is composed of a range of tangible and intangible resources, we did not incorporate household income (41). For household income to be informative as an index of SES, it should be examined in the context of other variables, such as number of adults and children in the family drawing on that income, regional cost of living, and number of caregiving adults in the family. Without these additional variables, family income poses a challenge to interpret. Therefore, parental educational attainment is a robust indicator of SES, regardless of these contextual factors, which aligns with other scholarly findings (42–44).

#### 2.3.5 Analytic *procedures*

All analyses were conducted in StataSE 17 (45). We used linear mixed-effect regression to test the longitudinal association between family conflict and adolescent alcohol expectancy (research question 1). Linear mixed-effect models capture correlation of data induced by having multiple members from the same family in the analysis. Thus, we included family membership as a random effect in models; family conflict and other covariates were all included as fixed effects. The moderating effects of race and ethnicity on the longitudinal association between family conflict and adolescent alcohol expectancy were tested using multiplicative interaction terms (research question 2). Specifically, we calculated product terms of family conflict with each of the binary race and ethnicity dummy variables discussed above. The moderation models were run separately for each race/ethnicity. The linear mixed-effect regression assumptions were checked: variance inflation factors (VIF), for multicollinearity (mean VIF = 1.10, see **Supplementary Table 1**), a two-way scatter plot to assess linearity for the independent and dependent variables (indicating a linear relationship for negative and positive alcohol expectancies), Shapiro–Wilk test for normality (non-normality detected, but the large sample size ensures robustness), and Breusch–Pagan test for heteroscedasticity (heteroscedasticity present for negative alcohol expectancies, p <.000, but not for positive alcohol expectancies, p <.489).

## 3 Results

### 3.1 Descriptive statistics

**Supplementary Table 2** displays the descriptive statistics for the variables used in the study. The mean values for the summary scores of negative and positive alcohol expectancies at year 3, family conflict at year 1, negative and positive alcohol expectancy at year 1, and family conflict at year 3 for the entire sample were 12.46, 8.51, 1.92, 12.08, 7.26, and 1.96, respectively. The demographics of the sample population were 58.11% White, 10.38% Black, 19.29% Hispanic, 2.25% Asian, 9.97% Other, and 52.72% male. Black adolescents had the highest mean score for family conflict at 2.29, and the lowest year 3 negative alcohol expectancy score at 11.66. Black and White adolescents were the only racial/ethnic groups with statistically significant correlations between the main variables of family conflict at baseline and alcohol expectancy at year 3. See **Supplementary Table 3** for the correlation matrix of the variables used in this study.

### 3.2 Regression analysis

First, we conducted two separate linear mixed-effect regressions predicting negative and positive alcohol expectancy from family conflict while controlling for the covariates (**Table 2**). The individual predictor of family conflict had significant and non-significant beta weights (*B*), based on p-value less than.05, when considered independently in predicting negative or positive alcohol expectancy: *B* = −0.040, *p* = 0.047, and *B* = 0.031, *p* = 0.199, respectively. See **Table 2** for the regression coefficients of each predictor.

**Table 2:** Results of linear mixed-effects regression examining family conflict and alcohol expectancy (AE).

Variables	*B*	SE	p-value
Negative AE at year 3			
Family conflict	−.040*	.020	.047
Sex	.158*	.066	.016
Age	.008*	.004	.074
Family SES	.240***	.023	.000
Negative AE at year 1	.171***	.011	.000
Family conflict at year 3	−.010	0.19	.604
Positive AE at year 3			
Family conflict	.031*	.024	.199
Sex	.15*	.079	.147
Age	.045***	.005	.000
Family SES	.107***	.027	.000
Positive AE at year 1	.311***	.013	.000
Family conflict at year 3	-.020*	.023	.388

B, beta-weights; SE, standard error; AE, alcohol expectancy; SES, socioeconomic status.

*p < 0.5, **p < 0.01, ***p < 0.001.

For each race/ethnicity separately, we ran linear mixed-effect regressions with multiplicative terms, predicting negative or positive alcohol expectancy (**Table 3**). The interaction multiplicative terms were significant for Black adolescents on negative alcohol expectancy and White adolescents on positive alcohol expectancy. The models indicated that the association between family conflict and negative alcohol expectancy was moderated by adolescents of Black race/ethnicity (*B* = −0.174, *p* = 0.003), and the association of family conflict and positive alcohol expectancy was moderated by adolescents of White race/ethnicity (*B* = 0.087, *p* = 0.046). The interaction was not significant for adolescents who identified as Hispanic, Asian, or Other.

**Table 3:** Results of linear mixed-effects regression examining family conflict and alcohol expectancy (AE) with multiplicative terms.

Variables	*B*	SE	p-value
Negative AE at year 3			
Family conflict	−.019*	.021	.368
Black	−.143*	.168	.396
Sex	.165*	.066	.012
Age	.008*	.004	.068
Family SES	.223***	.023	.000
Negative AE at year 1	.168***	.011	.000
Family conflict at year 3	−.007	.019	.700
Family conflict * Black	−.174**	.059	.003
Positive AE at year 3			
Family conflict	−.020	.035	.576
White	−.143*	.117	.220
Sex	.117*	.079	.140
Age	.045***	.005	.000
Family SES	.105***	.029	.000
Positive AE at year 1	.311***	.013	.000
Family conflict at year 3	−.021*	.023	.353
Family conflict * White	.087*	.044	.046

B, beta-weights; SE, standard error; AE, alcohol expectancy; SES, socioeconomic status. The interaction was not significant for adolescents who identified as Asian, Hispanic, or Other.

*p < 0.5, **p < 0.01, ***p < 0.001

For the significant interaction involving Black adolescents, the negative effect of family conflict on negative alcohol expectancy was significant for Black adolescents (*B* = −0.166, *p* = 0.033), but not for non-Black adolescents (β = −0.021, p = 0.313). For the significant interaction involving White adolescents, the positive effect of family conflict on positive alcohol expectancy was significant for White adolescents (β = 0.071, p = 0.023) but not for non-White adolescents (*B* = −0.022, *p* = 0.572). See **Table 4** for the regression coefficients of the product term analyses.

**Table 4:** Results of product term analyses for statistically significant multiplicative terms.

Variables	*B*	SE	p-value
Negative AE at year 3			
** For Black adolescents:**			
Family conflict at year 1	−.166*	.078	.033
Sex	.184*	.261	.481
Age	.007	.018	.705
Family SES	.427***	.088	.000
Negative AE at year 1	.229***	.038	.000
Family conflict at year 3	.003	.071	.971
** For non-Black adolescents:**			
Family conflict at year 1	−.021*	.021	.313
Sex	.163*	.067	.015
Age	.008*	.004	.088
Family SES	.198***	.023	.000
Negative AE at year 1	.159***	.011	.000
Family conflict at year 3	−.008	.020	.667
Positive AE at year 3			
** For White adolescents:**			
Family conflict at year 1	.071*	.031	.023
Sex	.115*	.101	.252
Age	.048***	.007	.000
Family SES	.115**	.043	.007
Positive AE at year 1	.333***	.016	.000
Family conflict at year 3	−.029*	.030	.320
** For non-White adolescents:**			
Family conflict at year 1	−.022	.038	.572
Sex	.126*	.127	.320
Age	.043***	.009	.000
Family SES	.103**	.040	.010
Positive AE at year 1	.279***	.021	.000
Family conflict at year 3	−.007	.036	.842

B, beta-weights; SE, standard error; AE, alcohol expectancy; SES, socioeconomic status.

*p < 0.5, **p < 0.01, ***p < 0.001

## 4 Discussion

The results of this study suggest that family conflict may be a risk factor for problematic alcohol expectancies for both Black and White adolescents. These findings are consistent with previous findings linking family conflict to adolescent alcohol use (21, 25–28, 46). These findings are also consistent with previous findings that implicated positive alcohol expectancies as a mechanism through which family conflict affects alcohol use in female American Indian adolescents (30). Although we tested the Black and White identities separately, our results indicated that family conflict may operate differently for Black and White adolescents in conferring the risk for problematic alcohol expectancies. For Black adolescents, higher family conflict predicts lower negative alcohol expectancy; however, for White adolescents, higher family conflict predicts higher positive alcohol expectancy.

Prevention and intervention efforts targeting family conflict may thus be relevant for different aspects of problematic alcohol expectancies in Black and White families. Specifically, in the presence of high family conflict, professionals supporting Black adolescent may consider paying close attention to, and where appropriate, boosting the adolescents’ negative alcohol expectancies. Professionals supporting White adolescent may consider paying close attention to and, where appropriate, challenging the adolescents’ positive alcohol expectancy.

Black adolescents reported higher levels of family conflict compared with adolescents of other ethnic and racial groups. This finding supported our first hypothesis based on the Minority Family Stress Model that adolescents from minoritized ethnic and racial backgrounds would report higher levels of family conflict than White adolescents. It is crucial to emphasize that this finding does not indicate that Black families are more dysfunctional than families of other racial or ethnic backgrounds. Rather, this racial/ethnic difference in family conflict likely points to other social contexts that uniquely affect Black families in the United States and were not explicitly considered in our model. Black families likely experience greater family stress than other families due to systemic and structural racism and discrimination that uniquely affect Black communities (47). This underscores the significance of addressing social context in understanding substance use and abuse in adolescents rather than individual or family-level factors.

Our study indicated that family conflict is worth further examination as a significant contributor to adolescent alcohol expectancies, with differences across racial/ethnic backgrounds. Family conflict is only one aspect of an adolescent’s social context, and researchers should consider other aspects, such as experiences of racial discrimination and racism, cultural values, or community and neighborhood factors (48–51). Building more complex, multivariate models of social context would advance the field toward a more nuanced understanding of the role of the social environment in adolescent risky behavior. For example, a recent ABCD dataset study by Trevino and colleagues modeled several aspects of the family environment in predicting genetic risk for alcohol use disorder (52).

To better understand the association between family conflict and alcohol expectancies, future studies should explore why family conflict may confer the risk for alcohol expectancies differently for Black and White adolescents. One hypothesis to explain this racial difference is that the function of alcohol consumption may vary culturally in Black and White families. Alcohol consumption may serve more as a coping mechanism within Black communities, whereas it may be more commonly associated with recreational activity in White communities (49). Additionally, lower negative alcohol expectancies are problematic for Black adolescents, as this may shift to higher positive alcohol expectancies, which acts as a precursor for alcohol initiation (53–55). Therefore, Black adolescents may be primed to retrieve lower negative expectancies, whereas White adolescents may be primed to retrieve more positive expectancies when thinking about alcohol. Cultural aspects of alcohol consumption for other racial/ethnic groups may also contribute to the association between family conflict and alcohol expectancies; however, a statistically significant relationship was not established for adolescents of Asian, Hispanic, or Other racial/ethnic backgrounds in our study. We may consider other cultural differences in parenting styles, family dynamics, or religious and spiritual beliefs to further explore the cultural context (21).

There is an established understanding that adolescence is a period of exponential brain development (56–58); however, the long-term effects of family conflict on the brain are not well-researched. Additionally, the National Institute on Drug Abuse (NIDA) is prioritizing research on how individual life experiences related to brain development and drug use trajectories (59). Thus, future studies should also consider how the functional and structural development of the brain mediates the development of alcohol expectancies and how stress from family conflict impacts brain development. For example, Hoffman et al. (60) explored the relationship of stress exposures, neurodevelopment, and health outcomes including substance use and addiction. Zhornitsky et al. (61, 62) found that positive alcohol expectancies were associated with functional connectivity such as thalamic activation and, thalamic-caudate connectivity to predict problematic alcohol use. Additionally, family conflict may be stressful for adolescents, which may interfere with the development of grey matter in the prefrontal cortex, resulting in impaired cognitive and emotional processes that impact decision-making and impulse control (63, 64). Establishing a better understanding of the brain mechanisms in the relationship between family conflict and alcohol expectancy could inform intervention and prevention strategies such as identifying brain markers that may be associated with the risk of problematic alcohol use, and specific therapies that may target those brain mechanisms to strengthen mechanisms related to decision-making and impulse control.

### 4.1 Limitations

It is essential to acknowledge the limitations of this study. Data on family conflict and alcohol expectancy were obtained from adolescents. Parents or guardians may have different views on family conflict and adolescents’ dispositions toward alcohol. This study did not include data on parent, guardian, family member, and/or adolescent alcohol use. We plan to include these data in future studies to assess the influence of alcohol use on alcohol expectancy, to build on previous research (65). Also, although the ABCD cohort is vast and representative of diverse regions and backgrounds, it may not be representative of the entire adolescent population due to selection and self-selection biases. ABCD participants were recruited from regions near ABCD study sites and may not reflect diversity across other geographic locations. Different sample sizes across racial groups may have differential effects that may have influenced the non-significant observed effects of the product term analyses. Furthermore, the five-level racial/ethnic construct may not capture the nuanced and multifaceted complexity of individual race/ethnicity. These findings may be an underrepresentation for individuals who identify as Asian, AIAN, NHPI, or mixed-race. Families who self-select to participate in prospective cohort studies that require considerable time and commitment may exhibit a higher level of functioning than families who do not participate. Finally, future studies could explore robust regression models to address heteroscedasticity and validate the results.

## 5 Conclusion

This longitudinal analysis showed that family conflict assessed at year 1 was associated with adolescent alcohol expectancies at year 3 follow-up for Black and White adolescents, but not adolescents of other racial and ethnic backgrounds. Specifically, family conflict was associated with lower negative alcohol expectancy for Black adolescents and higher positive alcohol expectancy for White adolescents. This study contributes to existing literature on social contexts and alcohol among adolescents by examining the interplay between family conflict, race, and alcohol expectancy in the ABCD study. The findings suggest that the association between family conflict and alcohol expectancy may be associated with adolescents’ racial or ethnic identity, with stronger effects observed among Black and White adolescents than adolescents who identified as Hispanic, Asian, or Other. This contributes to a better understanding of social factors that shape alcohol-related beliefs and highlights the need for targeted interventions that consider experiences of different racial and ethnic groups in promoting adolescent health.

## Data availability statement

The raw data supporting the conclusions of this article will be made available by the authors, without undue reservation.

## Ethics statement

Ethical review and approval was not required for the study on human participants in accordance with the local legislation and institutional requirements. Written informed consent from the patients/participants or patients/participants’ legal guardian/next of kin was not required to participate in this study in accordance with the national legislation and the institutional requirements.

## Author contributions

SB, MJ, WT, and MI contributed to conception and design of the study. WT and MI organized the database. MA, AP, HG, and NA provided technical expertise. SB performed the statistical analysis and wrote the first draft of the manuscript. MI supervised the overall project. All authors contributed to the article and approved the submitted version.
