# Supplementary material for: Prospective associations of family conflict with alcohol expectancies in the adolescent brain cognitive development study: effects of race and ethnicity
Source: Front Psychiatry. 2024 Mar 14;15:1250351. doi: 10.3389/fpsyt.2024.1250351 (PMC10973125; doi:10.3389/fpsyt.2024.1250351)
Supplement: Supplementary file 1 [file Table_1.docx]

Supplementary Material

Prospective Associations of Family Conflict with Alcohol Expectancies in the Adolescent Brain Cognitive Development Study: Effects of Racial and Ethnic Identity

**Skye C. Bristol*, Micah E. Johnson, Wesley K. Thompson, Matthew Albaugh, Alexandra Potter, Hugh Garavan, Nicholas Allgaier, Masha Y. Ivanova**

*** Correspondence:** Skye C. Bristol, [sbristol@usf.edu](mailto:sbristol@usf.edu)

# Supplementary Figures and Tables

| **Supplemental Table 1.** Variance Inflation Factor | |
| --- | --- |
| Family conflict at year 1 | 1.27 |
| Sex | 1.01 |
| Age (in months) | 1.01 |
| Family SES | 1.02 |
| Negative AE at year 1 | 1.01 |
| Positive AE at year 1 | 1.02 |
| Family conflict at year 3 | 1.26 |
| Mean VIF | 1.10 |

| **Supplemental Table 2. Descriptive statistics by race and ethnicity, mean (SD)** | | | | | | |
| --- | --- | --- | --- | --- | --- | --- |
|  | **Total** | **White** | **Black** | **Hispanic** | **Asian** | **Other** |
|  | **N = 6,231** | **n = 3,621** | **n = 647** | **n = 1,202** | **n = 140** | **n = 621** |
| Negative AE at year 3 | 12.46 (2.66) | 12.74 (2.37) | 11.66 (3.41) | 11.99 (2.92) | 12.75 (2.10) | 12.49 (2.62) |
| Positive AE at year 3 | 8.51 (3.22) | 8.62 (3.17) | 7.99 (3.39) | 8.24 (3.24) | 8.91 (2.89) | 8.86 (3.27) |
| Family conflict at year 1 | 1.92 (1.88) | 1.81 (1.87) | 2.29 (1.91) | 1.89 (1.81) | 1.56 (1.71) | 2.07 (1.96) |
| Sex |  |  |  |  |  |  |
| Male | 3,285 | 1,937 | 322 | 644 | 72 | 310 |
| Female | 2,946 | 1,684 | 325 | 558 | 68 | 311 |
| Age (in months) | 118.98 | 119.17 (7.52) | 118.88 (7.28) | 118.55 (7.55) | 119.58(7.83) | 118.88 (7.48) |
| Family SES |  |  |  |  |  |  |
| < HS Diploma | 302 | 28 | 48 | 201 | 1 | 24 |
| HS Diploma/GED | 535 | 167 | 125 | 195 | 3 | 45 |
| Some college | 971 | 458 | 157 | 230 | 6 | 120 |
| Associate | 801 | 416 | 114 | 199 | 1 | 71 |
| Bachelor | 1,916 | 1,359 | 109 | 225 | 44 | 179 |
| Post Graduate Degree | 1,698 | 1,192 | 91 | 149 | 85 | 181 |
| Negative AE at year 1 | 12.08 (3.06) | 12.30 (2.84) | 11.56 (3.47) | 11.80 (3.30) | 12.19 (2.65) | 12.19 (3.02) |
| Positive AE at year 1 | 7.26 (3.07) | 7.40 (3.06) | 7.05 (3.20) | 6.94 (2.98) | 7.38 (2.97) | 7.42 (3.06) |
| Family conflict at year 3 | 1.96 (1.92) | 1.91 (1.90) | 2.33 (2.04) | 1.94 (1.85) | 1.63 (1.83) | 2.01 (1.98) |
| *Note*. Frequency is reported for sex and family SES. All other characteristics are reported by mean for the overall sample and by each race/ethnicity. | | | | | | |

| **Supplemental table 3. Correlation Matrix** | | | | | | | | | | | | | | |
| --- | --- | --- | --- | --- | --- | --- | --- | --- | --- | --- | --- | --- | --- | --- |
| Variables | Negative AE year 3 | Positive AE year 3 | Family conflict year1 | White | Black | Hispanic | Asian | Other | Sex | Age | Family SES | Negative AE year 1 | Positive AE year 1 | Family conflict year 3 |
| Negative AE year 3 | 1.000 |  |  |  |  |  |  |  |  |  |  |  |  |  |
| Positive AE year 3 | -0.023* | 1.000 |  |  |  |  |  |  |  |  |  |  |  |  |
| Family conflict year 1 | -0.060*** | 0.019 | 1.000 |  |  |  |  |  |  |  |  |  |  |  |
| White | 0.095*** | 0.038*** | -0.073*** | 1.000 |  |  |  |  |  |  |  |  |  |  |
| Black | -0.054*** | -0.057*** | 0.078*** | -0.400*** | 1.000 |  |  |  |  |  |  |  |  |  |
| Hispanic | -0.079*** | -0.038*** | 0.019 | -0.573*** | -0.158*** | 1.000 |  |  |  |  |  |  |  |  |
| Asian | 0.004 | 0.020 | -0.022*** | -0.185*** | -0.051*** | -0.073*** | 1.000 |  |  |  |  |  |  |  |
| Other | -0.002 | 0.034*** | 0.028*** | -0.403*** | -0.112*** | -0.160*** | -0.051*** | 1.000 |  |  |  |  |  |  |
| Sex | 0.029** | 0.006 | -0.074*** | -0.017** | 0.018** | -0.008 | 0.005 | 0.017 | 1.000 |  |  |  |  |  |
| Age | 0.164*** | 0.078*** | -0.134*** | 0.365*** | -0.254*** | -0.275*** | 0.104*** | 0.012 | 0.000 | 1.000 |  |  |  |  |
| Family SES | 0.044*** | 0.131*** | -0.023** | 0.027*** | -0.006 | -0.029*** | 0.012 | -0.005 | -0.021** | 0.021** | 1.000 |  |  |  |
| Negative AE year 1 | 0.211*** | 0.005 | -0.043*** | 0.076*** | -0.069*** | -0.046*** | 0.005 | 0.012 | -0.027*** | 0.099*** | 0.067*** | 1.000 |  |  |
| Positive AE year 1 | -0.025* | 0.313*** | 0.067*** | 0.048*** | -0.028*** | -0.051*** | 0.006 | 0.018* | -0.023** | 0.071*** | 0.074*** | 0.014 | 1.000 |  |
| Family conflict year 3 | -0.048*** | -0.001 | 0.451*** | -0.032** | 0.065*** | -0.007 | -0.026** | 0.009 | -0.045*** | -0.099*** | -0.062*** | -0.039*** | 0.037*** | 1.000 |
| *Note.* Spearman rho correlation was used for ordinal variables of race and sex variables. Pearson’s R correlation was used for all other variables. AE = Alcohol Expectancy.  **** p<0.01, ** p<0.05, * p<0.1* | | | | | | | | | | | | | | |
